# Chinook and Coho salmon hybrids linked to habitat and climatic changes on Vancouver Island, British Columbia

**DOI:** 10.1002/ece3.8322

**Published:** 2021-11-11

**Authors:** H. Andres Araujo, William D. P. Duguid, Ruth Withler, Janine Supernault, Angela D. Schulze, Jessica L. Mckenzie, Kevin Pellett, Terry D. Beacham, Kim Jonsen, Anna Gummer

**Affiliations:** ^1^ Molecular Genetics Laboratory Pacific Biological Station Fisheries and Oceans Canada Nanaimo British Columbia Canada; ^2^ Department of Biology University of Victoria Victoria British Columbia Canada; ^3^ Department of Zoology The University of British Columbia Vancouver British Columbia Canada; ^4^ Ecosystems and Oceans Science South Coast Stock Assessment, Fisheries and Oceans Canada Nanaimo British Columbia Canada

**Keywords:** climate change, Cowichan River, hybridization, introgression, reproductive viability, salmonids, Strait of Georgia

## Abstract

Between 2013 and 2019, 63 presumed Chinook salmon *Oncorhynchus tshawytscha* sampled primarily in the Strait of Georgia (0.63% of total sample) were identified as potential Chinook–Coho (*Oncorhynchus kisutch*) hybrids by the presence of anomalous microsatellite genotypes. Their hybrid origin was confirmed by single nucleotide polymorphism amplification of two species‐specific amplicons. Mitochondrial DNA indicated that most of these fish resulted from the hybridization of Coho salmon females and Chinook salmon males. Although no diagnostic external features were identified, several individuals displayed an abnormal scale arrangement on the caudal peduncle. One hybrid juvenile examined for meristics exhibited a pyloric caeca count intermediate between published values for Chinook and Coho salmon. Most hybrids originated in the Cowichan River during the 2014 brood year. Their prevalence in the watershed is a naturally occurring event, likely exacerbated by prolonged low water levels which limit habitat and delay Chinook salmon spawning, in addition to the differential abundance of the parental species. This research is the first to document ongoing natural hybridization (Chinook–Coho salmon crosses) and link it to habitat and climatic changes, and includes the identification of eight F1 adults and two juvenile backcross or F2 hybrids. The potential negative impacts of hybridization, particularly in Coho salmon through potential introgression, warrant hybrid identification as an ecosystem monitoring tool within a survey program.

## Graphical abstract

From 2013 to 2019, 63 presumed Chinook salmon sampled primarily in the Strait of Georgia were identified as potential Chinook–Coho hybrids. Their origin was confirmed by nuclear and mitochondrial DNA techniques. Changes in the hydrological regime and spawning time suggest that the observed hybridization is a process resulting from freshwater habitat modification and climatic pressures.

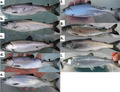

## 1 INTRODUCTION

Interspecific hybridization can be a major evolutionary factor that can increase adaptive capacity and generate new taxa (Abbott et al., 2013; Verspoor & Hammart, 1991) or impair fitness and extirpate or extinguish species (Allendorf et al., 2001; Rhymer & Simberloff, 1996). In salmonid fish, introgressive hybridization may result from limited barriers to reproduction between closely related species, the introduction of non‐native species, habitat alteration mediated by anthropogenic and climatic changes, or a combination of these factors (Castillo et al., 2008; Garcia de Leaniz & Verspoor, 1989; Hagen & Taylor, 2001; Muhlfeld et al., 2014; Scribner et al., 2001). Hybrids are important from an ecosystem monitoring perspective given that they tend to occur under circumstances that suggest rapid environmental change; determining their frequency and the conditions of their appearance could help scientists and managers take action to minimize negative ecological outcomes.

Pacific salmon occupy freshwater habitats subject to increasing human habitation, anthropogenic modification, water extraction, and climate change (Mantua et al., 2010). These factors place salmon at risk of “reverse speciation” and introgression, as environmental change weakens ecologically mediated reproductive isolation (Owens & Samuk, 2020). Negative consequences of introgression include outbreeding depression, lower performance in parental environment for traits such as feeding and predator avoidance (Wessel et al., 2006), early life history mortality (Bartley et al., 1990), and susceptibility to infectious diseases (Hedrick et al., 1987). In summary, highly hybridized populations have an elevated risk for extirpation or extinction (Rhymer & Simberloff, 1996).

Natural hybridization between Chinook (*Oncorhynchus tshawytscha*) and Coho salmon (*Oncorhynchus kisutch*) has been documented but is rare (Bartley et al., 1990; Chevassus, 1979; Johnson & Ringler, 1981). Its infrequency has been attributed to prezygotic isolation mechanisms that prevent viable development (Bartley et al., 1990). Utter et al. (1989) found no evidence of Chinook–Coho salmon hybrids in a Chinook salmon population structure study of 86 collection sites from the Babine River in British Columbia to the Sacramento River in California. However, Chinook–Coho F1 (first‐generation) hybrids have been detected in the wild (Bartley et al., 1990; Verspoor & Hammart, 1991) and viable and fertile hybrids are readily produced in laboratory settings (Argue & Dunham, 1999; Blanc & Chevassus, 1982; Foerster, 1935; Hedrick et al., 1987; Seeb et al., 1988; Smirnov, 1972). McKenzie et al. (2021) found no evidence for strong prezygotic isolation in noncompetitive heterospecific mating trials and in vitro fertilization experiments performed with Chinook and Coho salmon, although hybridization did not occur in mating trials in which a conspecific mate was present.

Few laboratory studies have addressed F2 (second generation) and backcrossed individuals resulting from the reproductive viability of F1 hybrids. Chevassus (1979) and references therein reported male fertility and moderate hatching success for F2 individuals. Devlin et al. (2001) reported excellent prehatch survival for eggs produced from an F1 male (from a Coho male by Chinook female cross) backcrossed to a Coho female. In natural environments, evidence is lacking on both the reproductive viability of hybrids beyond the F1 generation and the associated interspecific introgression that occurs between salmonids (Allendorf et al., 2001; Scribner et al., 2001).

Hybrids are often identified opportunistically in the context of other studies, based on atypical morphological characteristics or through genetic analysis. The latter is especially useful in the case of “cryptic” hybridization, which results in minimal morphological or meristic alteration (Allendorf et al., 2001; Scribner et al., 2001). In particular, the genotyping of codominant Mendelian markers at multiple loci, including those with alleles shared between the species, enables both the early detection of hybridization in closely related species and the estimation of proportions of first and second generation, as well as backcrosses to parental species (Anderson & Thompson, 2002). Inclusion of diagnostic single locus polymorphisms (SNPs) in multilocus genomic analyses facilitate hybrid detection in studies that may involve extensive sampling of individuals (Beacham & Wallace, 2020) and the application of many such SNPs enables the estimation of genome‐wide levels of introgression (Hohenlohe et al., 2011). Finally, examination of species‐specific mitochondrial DNA sequences can identify the maternal species involved in hybridization events (Karlsson et al., 2013), thus providing behavioral clues of the matings.

**FIGURE 1:**
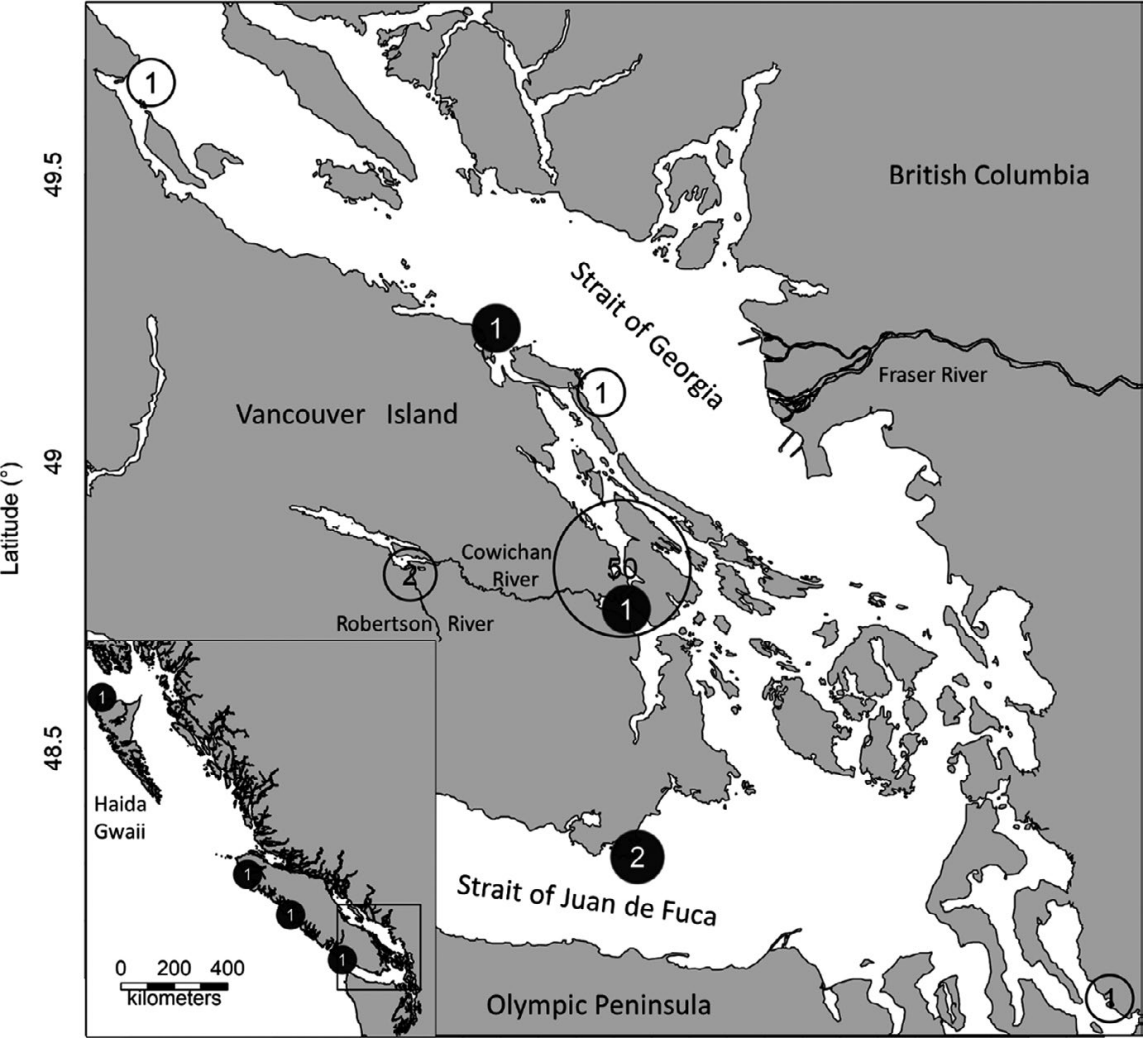
Locations of Chinook–Coho hybrid captures. Empty circles indicate juveniles, full circles adults. Survey details are provided in Table 2

The hybrids in this study were detected inadvertently by the presence of anomalous microsatellite genotypes during day‐to‐day genetic stock identification (GSI) analyses at the Fisheries and Oceans Canada – Molecular Genetics Laboratory (MGL) located at the Pacific Biological Station. GSI is used to assign mixed stock samples to a population of origin employing a baseline of genotypes and has been performed routinely in fisheries management and research contexts for four decades (e.g., Grant et al., 1980). In the current study, the hybrids were captured during the first summer of marine residence near the southern BC coast (Figure 1) as part of a tagging study to investigate survival of Cowichan River Chinook salmon (Pacific Salmon Foundation, 2017).

The Cowichan River drains a 930‐km^2^ watershed on southern Vancouver Island. Flow is partially controlled by a weir at the outlet of the Cowichan Lake. This system has been subject to extensive channel modifications, as well as surface and groundwater diversions. The Cowichan River has a recovering population of fall‐run Chinook salmon and supports a historically large run of Coho salmon, the current status of which is poorly monitored. Natural spawning of fall Chinook salmon is supplemented by a hatchery operated by the Cowichan Tribes with the support of Fisheries and Oceans Canada. The Cowichan Lake Salmon Enhancement Society (CLSES), a volunteer‐driven organization that rescues Coho salmon fry from becoming trapped by droughts during the summer months, also operates a small hatchery which supplements Chum salmon (*Oncorhynchus keta*).

The main objective of this study was to understand the biological and environmental processes behind the observed hybridization. To achieve this, we conducted a retrospective examination of the anomalous genotypes encountered in presumed Chinook salmon sampled primarily near the Cowichan River in the Strait of Georgia. We confirmed their hybrid status and determined if they had arisen through natural or hatchery spawning events. We applied molecular analyses of nuclear and mitochondrial DNA sequences to confirm the maternal species involved in hybridization, and estimated the numbers of potential parents and the proportions of first (F1) and second or higher generation (F2). We investigated if hybrids confirmed by molecular techniques exhibited consistent meristic traits that would allow rapid identification. We suggested the Cowichan River watershed as the predominant source of these hybrids and examined habitat and climatic alterations that have occurred in recent decades and their effects on spatiotemporal overlap spawning between Chinook and Coho salmon spawning.

## 2 METHODS

The samples in this study originated from a variety of sources, which prompted the creation of several databases (Table 1). Aspects such as number of individuals analyzed, category (e.g., hybrids vs. pure breeds), origin (e.g., surveys vs. hatchery origin), and the quantity and type of genetic markers used (e.g., microsatellites or SNPs) were employed depending on the purpose. Although not directly related to the genetic analyses, we include two sections on *morphology* and *hydrology* to examine meristic traits that can help screening Chinook–Coho hybrids and provide environmental clues about the cospawning of the two species involved. The following organizations provided the samples: Fisheries and Oceans Canada (DFO), the BC Conservation Foundation, the Cowichan Tribes, the CLSES, and the North West Indian Fisheries Commission in the United States.

**TABLE 1:** Databases employed in this study

Database	Individuals analyzed	Category	Individuals origin	Genetic markers used	Purpose
1	63	Chinook–Coho salmon hybrids	18 Surveys (see Table 2)	15 Microsatellites and five SNPs	Hybrid status identification
2	880	Pure breed Chinook	Hatchery broodstock	Five SNPs	Inspect the MGL genetic baseline for errors in broodstock identification, potentially leading to human‐induced hybridization
3	200	54 Hybrids (those with complete genotypes) +146 Chinook as control group	18 Surveys and hatchery broodstock, respectively	Six Microsatellites (common to both species)	Evaluate filial generation
4	576	31 Juvenile hybrids, five adult hybrids, and 540 juvenile Chinook Cowichan origin (all from brood year 2014)	18 Surveys (Table 2) 540 Juvenile Chinook from juvenile marine tagging surveys 2015	Six microsatellites (common to both species)	Examine the family structure of the hybrid group
5	103	63 Chinook–Coho salmon hybrids, 20 pure Coho, 20 pure Chinook	18 Surveys (see Table 2) MGL genetic baseline	qPCR panel (Table 4)	Determine the maternal species involved in the hybridization

### 2.1 Study subjects

Between 2013 and 2017, 62 presumed Chinook salmon sampled in coastal waters of British Columbia by a variety of surveys were identified as potential Chinook–Coho salmon hybrids (Table 2; Figure 1).

**TABLE 2:** Numbers and percentages of pure Chinook and Chinook–Coho hybrids in the surveys and in relation to Cowichan Chinook identified by GSI

Survey	Year	No. of samples (presumed Chinook)	Life stage	No. of hybrids	Cowichan Chinook by GSI	% Cowichan Chinook by GSI	% Hybrids in total sample	% Hybrids in relation to Cowichan Chinook by GSI
DFO Trawl	2013	783	J	1	29	3.70	0.13	3.45
DFO Trawl	2014	794	J	1	12	1.51	0.13	8.33
DFO Area 121 Recreational	2015	72	A	1	0	0.00	1.39	NA
NWIFC Puget Sound	2015	1698	J	1	0	0.00	0.06	NA
DFO Area 19/20 Recreational	2016	222	A	1	0	0.00	0.45	NA
DFO Area 1 Recreational	2017	552	A	1	0	0.00	0.18	NA
DFO Area 125 Recreational	2017	37	A	1	1	2.70	2.70	100
DFO Area 17 Recreational	2017	405	A	1	44	10.86	0.25	2.27
DFO Area 19/20 Recreational	2017	211	A	1	6	2.84	0.47	16.67
DFO Area 27 Recreational	2017	285	A	1	0	0.00	0.35	NA
DFO Seine	2015	276	J	7	231	83.70	2.54	3.03
BCCF Marine Tagging	2015	1222	J	22	447	36.58	1.80	4.92
DFO Seine	2016	298	J	2	287	96.31	0.67	0.70
BCCF Marine Tagging	2016	1234	J	7	1039	84.20	0.57	0.67
CLSES Robertson R	2016	82	J	2	77	93.90	2.44	2.60
DFO Seine	2017	134	J	2	118	88.06	1.49	1.69
BCCF Marine Tagging	2017	1697	J	10	1250	73.66	0.59	0.80
BCCF Marine Tagging	2019	1	A	1	NA	NA	NA	NA

Note

Aggregations are presented in Figure 1. Surveys near the Cowichan River estuary in gray.

Abbreviations: A, Adult; J, Juvenile.

These fish exhibited unusual genotypes and “stutter patterns” or peak morphologies observed during DNA amplification of 15 microsatellite loci (Table 3), which are used routinely to perform GSI in the identification of mixed stock samples. The hybrid samples were observed from a total mixed stock sample of 10,003 fish (0.63%). The majority of the hybrid samples were juvenile fish captured by microtrolling (Duguid & Juanes, 2017) in marine waters near the Cowichan River as part of a passive integrated transponder (PIT) tagging study to investigate survival of Cowichan River Chinook salmon (Pacific Salmon Foundation, 2017). Additional samples were collected in routine juvenile surveys in the Strait of Georgia by Fisheries and Oceans Canada and in creel monitoring in the same area and elsewhere in British Columbia. Two juveniles (fry) were collected within the Cowichan drainage during a fry salvage operation by the CLSES. An additional sample (# 63) was an adult tagged in marine waters near Cowichan Bay in September 2019 during a project to investigate pinniped predation on returning Cowichan River Chinook salmon. All hybrid samples were screened for coded wire tags (CWTs) or adipose fin clips that would indicate hatchery origin. More than 90% of Chinook salmon juveniles released from the Cowichan River hatchery in the years pertinent to this study had been fin‐clipped as part of the Salish Sea Marine Survival Project (K. Pellett, unpublished data).

**TABLE 3:** Microsatellite locus suite run for Chinook salmon in the DFO MGL and allelic size ranges found in Cowichan River Chinook salmon for each locus, as well as ranges in Coho salmon and putative Chinook–Coho salmon hybrids

Loci	Cowichan Chinook salmon allelic size range	Coho salmon allelic size range (also observed in hybrids)
Ots9^a^	104–116	98–102
Ogo4	140–180	112–138
Ots213^a^	195–317	163–293
Ots104^a^	183–295	351–487
Ots107	184–308	200–360
Oki100	210–304	222–395
Omy325^a^	84–116	118–160
Ots101	159–290	124–242
Ots2	138–176	136–142
Ogo2^a^	213–238	240–260
Ots100	219–436	227–409
Ots211^a^	208–334	400–412
Ssa197	140–290	230–262
Oke4	219–239, 243–265	239–249
Ots201b	140–343	No amplification

^a^ Loci included in the family analysis of the YC14 hybrids.

### 2.2 Hybrid identification microsatellites

Hybrid fish were detected in microsatellite analyses of presumptive Chinook salmon with a standard set of 15 loci for the species (Table 3). Most samples had been collected nonlethally and consisted of fin clips affixed to Whatman paper or preserved in 95% undenatured ethanol or scale samples on gummed scale books. DNA was extracted using a chelex resin protocol (Small et al., 1998) or a Promega Wizard SV96 Genomic DNA Purification System (Promega Corporation). The PCR fragments were size fractionated using an ABI 3730 capillary DNA sequencer for the set of microsatellites mentioned earlier; the genotypes were scored with either ABI Genotyper version 2.5 or ABI GeneMapper version 3.0 (equipment and software by Applied Biosystems, Inc. [ABI]).

Hybrid fish were identified by the presence of both Chinook and Coho salmon alleles, which presented anomalous peak morphologies among the Chinook salmon microsatellites used in their original screening (out of the allelic range expected for the species and presenting alleles corresponding to Coho salmon instead; see Table 3). In addition, Coho and Chinook salmon have distinctive 2‐ and 4‐bp “stutter” patterns, though with overlapping allele size ranges at the Oki100 locus. Many hybrids were initially recognized by the presence of one allele with the Coho 2‐bp stutter pattern. Because of their interspecific nature, hybrid fish could not be assigned to a population of origin by traditional GSI methods which require a baseline of genotypes from pure species individuals.

Pure species Coho salmon were not genotyped with the Chinook panel of 15 microsatellites as a control group because this has been done multiple times during regular GSI analyses at the DFO MGL, which results in informative allelic frequencies for the set of shared six loci used in this study, but “out of range” (e.g., allelic frequencies not available) for the set of nonshared loci. As the hybrid samples were detected using the microsatellite panel (used in day‐to‐day operations in the MGL as the default GSI method), and given the small number of markers, we added a SNP panel to validate their hybrid identification.

### 2.3 SNP species ID

The SNP panel is composed of two amplicons and five diagnostic SNPs. DNA amplicons OkiOts_120255 and Oki_RAD41030 have SNP sites fixed for alternate base pairs in Chinook and Coho salmon (Beacham & Wallace, 2020). OkiOts_120255 has a forward primer sequence of TGGAGTTGACAAAACATCCGATGTC and a reverse primer sequence of CCAGCAGACAGTCATCCTAAAAGAAA (Starks et al., 2016). The forward and reverse primers for Oki_RAD41030 are GGCTGGCTGAGCCTGGTCT and GCACTTTAGCTCTGCAATGCAGCT (Campbell et al., 2017). DNA from the suspected hybrids was loaded on a P1 chip v3 and run consecutively on the Ion Chef, and subsequently loaded on an Ion Torrent Proton sequencer (both machines by Thermo Fisher Scientific, Inc., 2019). Genotypes at one diagnostic position in OkiOts_120255 SNP: 113 (Reference = A, Variant = C) and four positions in Oki_RAD41030: 45 (TC), 51(CG), 195 (GA), and 198 (TG) were called via Proton software Variant Caller^®^. We classified a fish as a validated hybrid if one or both SNP amplicons were heterozygous for the Coho and Chinook salmon haplotypes. After the SNP panel had been applied to the putative hybrids, it was used to analyze 880 Cowichan River Chinook individuals for the presence of Coho or hybrid Chinook–Coho salmon genotypes (Table 1, No. 2). These individuals had been selected for broodstock at the Cowichan River hatchery from 2013 to 2017. The hatchery had submitted the samples for genetic analyses to become part of the MGL Chinook salmon baseline in a program to identify hatchery‐reared salmon based on population and individual identification through parentage‐based tagging and GSI. The main purpose of this procedure was to inspect the MGL genetic baseline for errors in broodstock identification, potentially suggesting human‐induced hybridization.

### 2.4 Filial generation and family structure

A subset of six microsatellite loci that amplified well in both species and had little or no overlap in allele size ranges (Table 3) was used to evaluate filial generation. In total, 54 suspected hybrids with complete genotypes were analyzed with this set, along with 146 pure Cowichan Chinook salmon as a control group (Table 1, No. 3). We employed the software package NewHybrids (Anderson & Thompson, 2002) to determine the most probable identity (F1, F2, backcross to Chinook, or backcross to Coho salmon). It is important to clarify that although NewHybrids has the ability to be distinguished between F2s and backcrosses, it is difficult to assign an individual to either category with certainty, and the margin of error may be too large to reach a definitive conclusion without sufficient loci showing extreme differences in allele frequencies (Anderson & Thompson, 2002), which is not the case for the sister species involved in the hybridization. The analysis was conducted for two million sweeps, without the training option indicating pure individuals of each species and assigning posterior probabilities of filial generation in hybrids.

A similar dataset (Table 1, No. 4) with the same six‐locus genotypes was used to examine the family structure of the hybrid group, which consisted of 540 juvenile Chinook salmon along with 31 juvenile hybrids that matched the same cohort (brood year 2014) captured in 2015 during surveys in marine waters near the Cowichan River (Table 2). We included five adults captured in 2017 that could have been produced from the 2014 parent's brood year, irrespective of their capture location. The rationale for this choice was to explore if any adults caught elsewhere may have originated in the Cowichan watershed. The pure species juvenile Chinook captured near the mouth of the Cowichan River were analyzed by GSI which confirmed their probabilistic allocation to that watershed (Table 2). We employed Colony 2.0.6.5 (Jones & Wang, 2010) to identify sibling relationships in the absence of parental genotypes. The chosen method assumed polygamy in both parents and applied *full likelihood* without parental genotypes and an error rate of 0.01. An analysis of the hybrid fish alone allowed us to estimate the number of Chinook and Coho salmon (or F1 hybrid) parents involved in their production and simulate their genotypes. In addition, we investigated if the Chinook salmon parents involved in the hybridization also produced purebred progeny with conspecific mates using the 540 juvenile samples.

### 2.5 Mitochondrial DNA

We employed mitochondrial DNA analyses to determine the maternal species involved in the hybridization and obtain behavioral clues of the matings. We conducted qPCR of the cytochrome c oxidase subunit 1 in Chinook salmon (Laramie et al., 2015) and of cytochrome b in Coho salmon qPCR assay (Pilliod & Laramie, 2016) on each hybrid fish, in addition 40 control samples of pure Coho and Chinook salmon (20 of each) were added. Amplification was conducted with Taqman probes labeled with 6FAM at the 5′ end and a minor groove binding nonfluorescent quencher at the 3′ end (MGB‐NFQ, Life Technologies). These Coho and Chinook salmon assays amplify in other salmonids at a reduced sensitivity (>1/25), so it is necessary to run both assays for complete maternal species identification. Additionally, we developed a Chinook salmon cytochrome b qPCR assay to confirm the results. The qPCR panel is presented in Table 4.

**TABLE 4:** qPCR assay details including primer and probe sequences for each species, assay length, and reference

Species	Gene	Symbol	Name	Sequence (5′–3′)	Assay length (bp)	Publication	Slope	Intercept	Efficiency	*R* ^2^
All salmon	Si:dkey‐78d16.1 protein [Danio rerio]	78d16	HKG_78d16_F	GTCAAGACTGGAGGCTCAGAG	102	Miller et al. (2016)	−3.26	27.39	1.02	0.97
			HKG_78d16_R	GATCAAGCCCCAGAAGTGTTTG						
			HKG_78d16_P	AAGGTGATTCCCTCGCCGTCCGA						
*Oncorhynchus kisutch*(Coho)	Cytochrome b	Cytb	Onki_Cytb_F	CCTTGGTGGCGGATATACTTATCTTA	114	Pilliod and Laramie (2016)	−3.48	21.30	0.94	0.97
			Onki_Cytb_R	GAACTAGGAAGATGGCGAAGTAGATC						
			Onki_Cytb_P	TGGAACACCCATTCAT						
*Oncorhynchus tshawytscha* (Chinook)	Cytochrome c oxidase subunit 1 region	COI	Onts_COI_F	CTGGCACMGGGTGAACAGTCTACC	90	Laramie et al. (2015)	−3.41	20.74	0.97	0.96
			Onts_COI_R	AATGAAGGGAGAAGATCGTYAGATCA						
			Onts_COI_P	CTCCTGCGTGGGCTAG						
*Oncorhynchus tshawytscha* (Chinook)	Cytochrome b	Cytb	Onts_Cytb_F	ATATACATATCGCCCGAGGACTTT	80	Miller et al. (Unpublished data)	−3.40	21.20	0.97	0.95
			Onts_Cytb_R	AAGTAGAAGTACCACCCCAATATTTCA						
			Onts_Cytb_P	TTATGGCTCTTACCTCTACAAA						

Note

qPCR assay performance details on the ABI7900 instrument including slope, intercept, efficiency, and *R* ^2^.

Reactions for qPCR analysis consisted of 12 μl total volume. qPCR analyses were conducted in 384‐well optical plates on a Life Technologies 7900 Real‐time qPCR System (Thermo Fisher Scientific, Inc.). All reactions used thermocycler conditions on standard mode: 2‐min warm up at 50°C, 10‐min initial heat activation at 95°C, followed by 40 cycles of 15 s denaturation at 95°C, and 60 s annealing/extension at 60°C. Data at each stage are available on request. The results for the entire qPCR assay are available as supplementary material (Appendix Table S1).

### 2.6 Morphology

Most hybrids were identified by genetic analyses after the physical specimens were no longer available for inspection, having been released as part of tagging studies or consumed by fishers. However, photographs were available for six juvenile hybrids captured and tagged as part of the Cowichan Chinook PIT tagging study and for the adult fish captured as part of the pinniped predation study in 2019 (Table 2). A single hybrid juvenile captured during the Cowichan PIT tagging in 2017 (#62 in Table 5) was retained and dissected in the laboratory for counts of pyloric caeca and branchiostegal rays.

**TABLE 5:** Hybrid individuals surveyed in the study including the surveys in which they were encountered, year, life stage, and other features results

Fish #	Survey	Year	Life stage	MtDNA	Generation	Used in generation analyses (Table 1, No. 3)	Used in family structure (Table 1, No. 4)	Confirmed by SNPs
1	DFO Trawl	2013	J	Coho	F1	Y		Y
2	DFO Trawl	2014	J	Coho	NA			
3	NWIFC Puget Sound	2015	J	No result	NA			
4	DFO Area 121 Rec.	2015	A	Coho	F1	Y		Y
5	DFO Seine	2015	J	Coho	F1	Y	Y	Y
6	DFO Seine	2015	J	Coho	F1	Y	Y	Y
7	DFO Seine	2015	J	Coho	F1	Y	Y	Y
8	DFO Seine	2015	J	Coho	F1	Y	Y	Y
9	DFO Seine	2015	J	Coho	F1	Y	Y	Y
10	DFO Seine	2015	J	Coho	F1	Y	Y	Y
11	DFO Seine	2015	J	Coho	F2^a^	Y	Y	Y
12	BCCF Marine Tagging	2015	J	Coho	F1	Y	Y	Y
13	BCCF Marine Tagging	2015	J	Coho	F1	Y	Y	Y
14	BCCF Marine Tagging	2015	J	Coho	F1	Y	Y	Y
15	BCCF Marine Tagging	2015	J	Coho	F1	Y	Y	Y
16	BCCF Marine Tagging	2015	J	Coho	F1	Y	Y	Y
17	BCCF Marine Tagging	2015	J	Coho	F2^a^	Y	Y	Y
18	BCCF Marine Tagging	2015	J	Coho	F1	Y	Y	Y
19	BCCF Marine Tagging	2015	J	Coho	F1	Y		Y
20	BCCF Marine Tagging	2015	J	Coho	F1	Y	Y	Y
21	BCCF Marine Tagging	2015	J	Coho	F1	Y	Y	Y
22	BCCF Marine Tagging	2015	J	No result	F1	Y	Y	Y
23	BCCF Marine Tagging	2015	J	No result	F1	Y	Y	Y
24	BCCF Marine Tagging	2015	J	No result	F1	Y	Y	Y
25	BCCF Marine Tagging	2015	J	No result	F1	Y	Y	Y
26	BCCF Marine Tagging	2015	J	No result	F1	Y	Y	
27	BCCF Marine Tagging	2015	J	No result	F1	Y	Y	Y
28	BCCF Marine Tagging	2015	J	No result	F1	Y	Y	Y
29	BCCF Marine Tagging	2015	J	No result	F1	Y	Y	Y
30	BCCF Marine Tagging	2015	J	No result	F1	Y	Y	Y
31	BCCF Marine Tagging	2015	J	No result	F1	Y	Y	Y
32	BCCF Marine Tagging	2015	J	No result	F1	Y	Y	
33	BCCF Marine Tagging	2015	J	No result	F1	Y	Y	Y
34	CLSES Robertson R.	2016	J	Chinook	F1	Y	Y	
35	CLSES Robertson R.	2016	J	Chinook	F1	Y	Y	
36	DFO Area 19/20 Rec.	2016	A	Ambiguous	F1	Y		Y
37	DFO Seine	2016	J	Coho	F1	Y		Y
38	DFO Seine	2016	J	Coho	F1	Y		Y
39	BCCF Marine Tagging	2016	J	Coho	F1	Y		
40	BCCF Marine Tagging	2016	J	Coho	F1	Y		Y
41	BCCF Marine Tagging	2016	J	Coho	F1	Y		Y
42	BCCF Marine Tagging	2016	J	Coho	NA			
43	BCCF Marine Tagging	2016	J	Coho – low detection	F1	Y		Y
44	BCCF Marine Tagging	2016	J	No result	F1	Y		
45	BCCF Marine Tagging	2016	J	No result	F1	Y		
46	DFO Area 1 Rec.	2017	A	Coho	F1	Y	Y	Y
47	DFO Area 125 Rec.	2017	A	Coho	NA		Y	Y
48	DFO Area 17 Rec.	2017	A	Coho	F1	Y	Y	Y
49	DFO Area 19/20 Rec.	2017	A	Coho	NA		Y	Y
50	DFO Area 27 Rec.	2017	A	Coho	NA		Y	Y
51	DFO Seine	2017	J	No result	F1	Y		
52	DFO Seine	2017	J	No result	F1	Y		
53	BCCF Marine Tagging	2017	J	Coho	F1	Y		Y
54	BCCF Marine Tagging	2017	J	Coho	F1	Y		Y
55	BCCF Marine Tagging	2017	J	Coho	F1	Y		Y
56	BCCF Marine Tagging	2017	J	Coho	F1	Y		Y
57	BCCF Marine Tagging	2017	J	Coho	F1	Y		Y
58	BCCF Marine Tagging	2017	J	Coho	F1	Y		Y
59	BCCF Marine Tagging	2017	J	Coho	F1	Y		Y
60	BCCF Marine Tagging	2017	J	Coho	NA			Y
61	BCCF Marine Tagging	2017	J	Coho	NA			Y
62	BCCF Marine Tagging	2017	J	Coho	F1	Y		
63	BCCF Marine Tagging	2019	A	No result	NA		Y	Y

^a^ Individuals that may be second generation F2 or backcross.

### 2.7 Hydrology and spawning

We looked at relationship between high and low flows and the timing of spawning in the Cowichan River for the species involved in the hybridization. Prespawning salmon (particularly those that enter the rivers in the late summer months) tend to wait until higher flow events facilitate their passage, obtain optimal temperature, and reach adequate dissolved oxygen levels (Bjorn & Reiser, 1991). This information allowed us to make inferences about the amount of habitat available and explore potential delays in the time of spawning, which provides clues on the convergence of the two species involved in the hybridization, with Chinook salmon historically spawning in the late summer and Coho salmon in the fall.

We obtained monthly discharge data from the Cowichan River measured downstream of lake outlet (Station Number 08HA002) from the Water Survey of Canada (2019) from 1960 to 2018 (postweir installation period) to explore the relationship between river discharge, abundance, and spawning migration time. Abundance and spawning migration time data were provided by the South Coast Stock Assessment Section captured at the fence operated by Fisheries and Oceans Canada. We estimated the weighted average Julian day of Chinook salmon counts by year, the Julian day where the peak of the migration occurs, and the mean run Julian day considering the beginning and end of the run independent of the number of counts.

## 3 RESULTS

Table 2 specifies numbers and percentages of pure Chinook and Chinook–Coho sampled in the marine environment and in relation to total samples identified by GSI. These results are not meant to be interpreted as hybridization rates, given the variable sample size and that these surveys were not originally designed to investigate hybrid occurrence. The intention of this information is to put the observed hybridization in context and present a general idea of its frequency: (1) independently of origin and (2) assuming that hybrids belong entirely to the cohorts originating in the Cowichan River. GSI results are probabilistic and thus the percentages presented in Table 2 may be subject to biological interpretation (e.g., the composition of the mixture employed and the baseline used in the analyses). Putting this caveat aside and considering only those surveys near the Cowichan watershed, the proportion of hybrid fish in the sample ranged between 0.57% when considering hybrids independent of the origin and 4.92% when assuming that hybrids originated in the Cowichan River.

### 3.1 Hybrid identification with microsatellites

All the 63 fish that were originally detected as potential hybrids in the GSI microsatellite analyses were confirmed as being the offspring of Chinook and Coho salmon at some or all of the loci surveyed. In the 54 hybrids with complete genotypes for the subset of six loci used in subsequent analyses (Table 1, No. 3), the Chinook salmon alleles carried by the hybrid progeny were those characteristic of the Cowichan Chinook salmon population, and ranged in number from 5 to 19 among loci. Alleles unambiguously identifiable as of Coho salmon origin in the hybrids ranged from 2 to 19 among loci. Table 5 provides a comprehensive summary of the characteristics of each identified hybrid. All hybrids in this study were likely the product of natural spawning, as none had a clipped adipose fin. Of the 880 Chinook salmon broodstock from the Cowichan River hatchery from 2013 to 2017 (Table 1, No. 3), no hybrids or Coho salmon were identified, further suggesting that hatchery propagation was not the source of the hybridization.

### 3.2 SNP species ID

Of the 63 hybrids, 50 were successfully amplified at the species‐specific SNP amplicons (Appendix Table S2). All 50 were heterozygous for a Chinook and Coho haplotype at both SNP loci, confirming these as the parental species involved in the hybridization and consistent with all being F1 or higher order (F2 or backcross) hybrid individuals. Of these, eight were adult samples caught in 2015, 2016, 2017, and 2019 in recreational fisheries from the southeast and west coast of Vancouver Island. The 42 validated juvenile samples were caught within the Strait of Georgia in the Fisheries and Oceans (DFO) seine and trawl surveys, the juvenile marine tagging studies in 2013, 2015, 2016 and 2017, and fry samples from the Robertson River within the Cowichan watershed in 2015.

### 3.3 Filial generation and family structure

In the NewHybrids analysis (Table 1, No. 3), all the 146 controls were associated with high posterior probabilities as belonging to the purebred Chinook salmon, while all the 54 presumed hybrids assigned to the hybrid category. All but two hybrids had high posterior probabilities of being F1 individuals (>0.98). The remaining two hybrid individuals 11 and 17 (Table 5) did not assign to the F1 category, instead these juveniles displayed moderate probabilities (0.59 and 0.52, respectively) of being Chinook backcross individuals (i.e., arising from a F1 hybrid mated with a Chinook salmon). In addition, these two individuals displayed 0.30 and 0.31 probabilities, respectively, of being F2 individuals arising from a F1 by F1 matings.

The colony analysis of family structure in the hybrid fish (Table 1, No. 4) indicated that they were the progeny of 19 Chinook salmon parents mated with 16 Coho (or F1) salmon. With respect to the 576 fish sampled in the study and assumed to belong to the year class arising from spawning in the Cowichan River watershed in 2014 (540 juvenile and five adult Chinook plus 31 hybrids), the analysis indicated that approximately 205 parents that included both sexes were responsible for the sample. Pure species Chinook salmon full‐sibling family sizes ranged from 1 to 15 and half‐sibling family sizes ranged from 2 to 18. Of the 19 Chinook salmon parents that produced hybrid progeny, six also produced purebred progeny with conspecific mates. Three of the five adult hybrids that may have originated from the 2014 year class, shared one of the “phantom” parents (for which genotypes are deduced in colony from those of their progeny) with hybrid juveniles, yielding inconclusive results.

### 3.4 Mitochondrial DNA

Mitochondrial DNA amplification was successful for 45 hybrid fish (Tables 1, No. 5 and 4). The cytochrome b for Coho salmon amplified in 41 individuals indicated that these were the offspring of Coho females. Two of the remaining four were the offspring of the Chinook salmon maternal line and the remaining two were ambiguous or presented low resolution. The hybrid individuals of the Chinook salmon maternal origin were two juveniles sampled as fry in the Robertson River tributary of Cowichan Lake and identified as full‐siblings in the colony analysis earlier. All other hybrids for which mtDNA amplification was successful were the result of Coho or F1 females mating with Chinook or F1 males. None of the negative controls amplified.

### 3.5 Morphology

**FIGURE 2:**
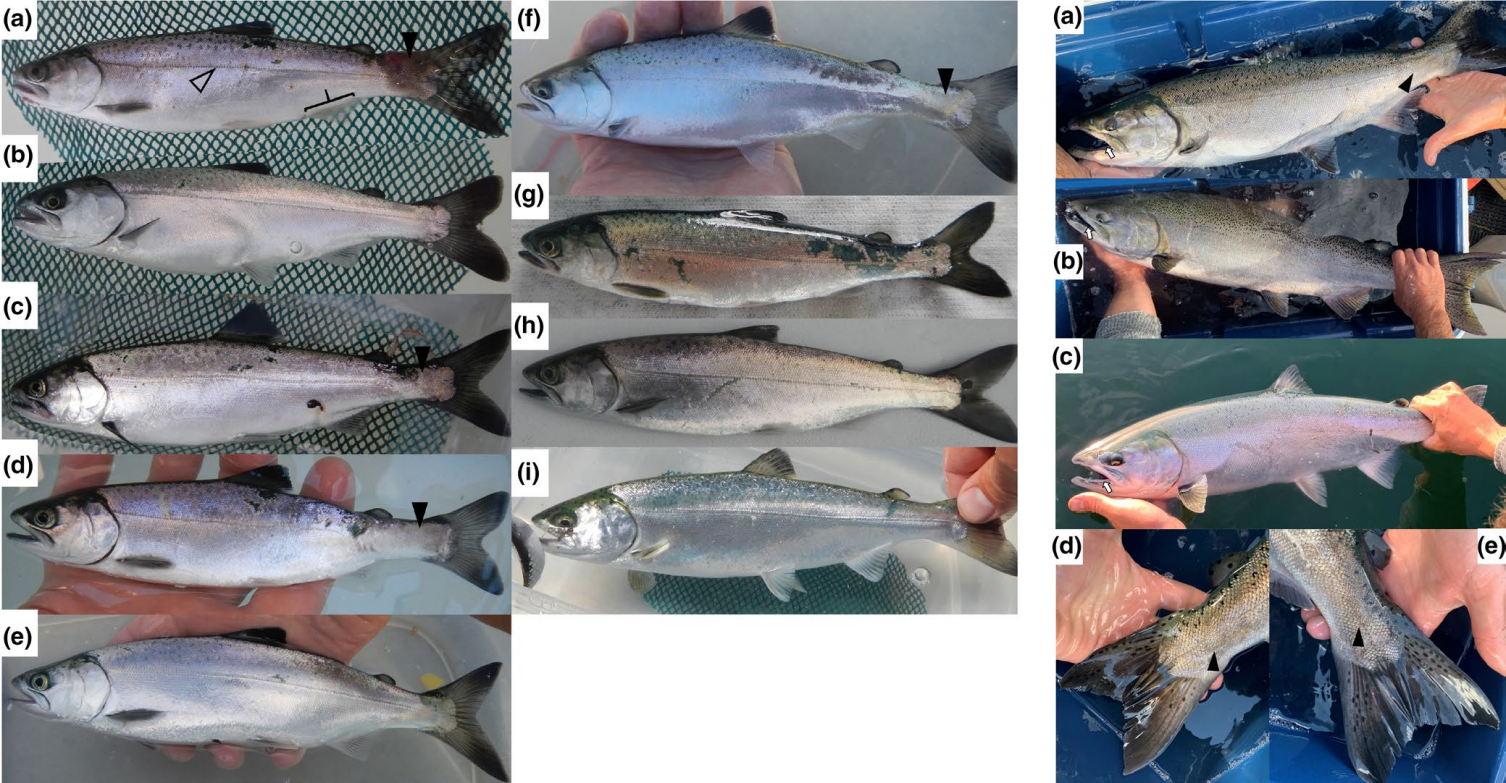
External features of juvenile and adult Chinook and Coho salmon hybrids in comparison to the parent species. 2.1: Hybrid Chinook–Coho salmon captured by microtrolling in 2015 (a–d), 2016 (e–f), and 2017 (g), compared to juvenile Cowichan River origin Chinook Salmon (h) and Coho salmon (i) captured during the same surveys. Four of seven photographed hybrids (a, c, d, and f) exhibited abnormal scale arrangement on the caudal peduncle (indicated by a filled triangle) in which the lateral line (indicated by the open triangle in (a)) disappeared at the caudal end. The {in (a) indicates the location of the base anal fin. 2.2: Adult hybrid Chinook–Coho salmon (a) captured at the entrance to Cowichan Bay on September 24, 2019 in comparison to Chinook (b) and Coho (c) salmon captured as part of the same study. Both sides of the caudal peduncle of hybrid (d, e) exhibited abnormal scale arrangement (filled triangles) and interruption of the lateral line. The gums (indicated by arrows) of the hybrid were intermediate in color between the black of the Chinook and white of the Coho salmon

Juvenile hybrids captured in the ocean exhibited a body morphology and coloration intermediate between Coho and Chinook salmon, but did not exhibit diagnostic external differences from Chinook salmon (Figure 2). The anal fin – for which the distal end of the most anterior ray will generally extend past the base of the most posterior ray when flattened against the body in Coho salmon, but not in Chinook salmon – had a morphology intermediate between the two species.

Four of the six juvenile hybrids for which photographs were available exhibited disordered arrangement of scales on the caudal peduncle, with the lateral line either deflecting or disappearing at the caudal end. Prior to initial identification of hybrids using microsatellites, no individuals were flagged as hybrids based on external morphology. Nevertheless, hybrids may be detected visually if their presence is expected, such as the adult salmon caught in 2019 (Fish 63, Table 5; Figure 2,2a,d,e), which was identified first visually by the overall body shape intermediate between a Chinook salmon and Coho salmon and exhibiting gum coloration intermediate between the black of Chinook salmon and the white of Coho salmon. The caudal peduncle also exhibited disordered scale arrangement on both sides, including the complete disappearance of the lateral line.

One juvenile hybrid (136 mm NFL) captured in 2017 and identified in the field as a hybrid based on morphology (Fish 62, Table 5; Figure 2,1g) was lethally sampled and examined in the laboratory. This individual did not exhibit abnormality of the caudal peduncle. The most anterior ray of the anal fin did not reach the attachment point of the most posterior ray when folded against the body. The branchiostegal count was 16, while the pyloric caeca count was 95. A Cowichan River origin Chinook salmon sampled lethally on the same day (141 mm NFL) had a branchiostegal count of 16 and a pyloric caeca count of 137.

### 3.6 Hydrology and spawning

**FIGURE 3:**
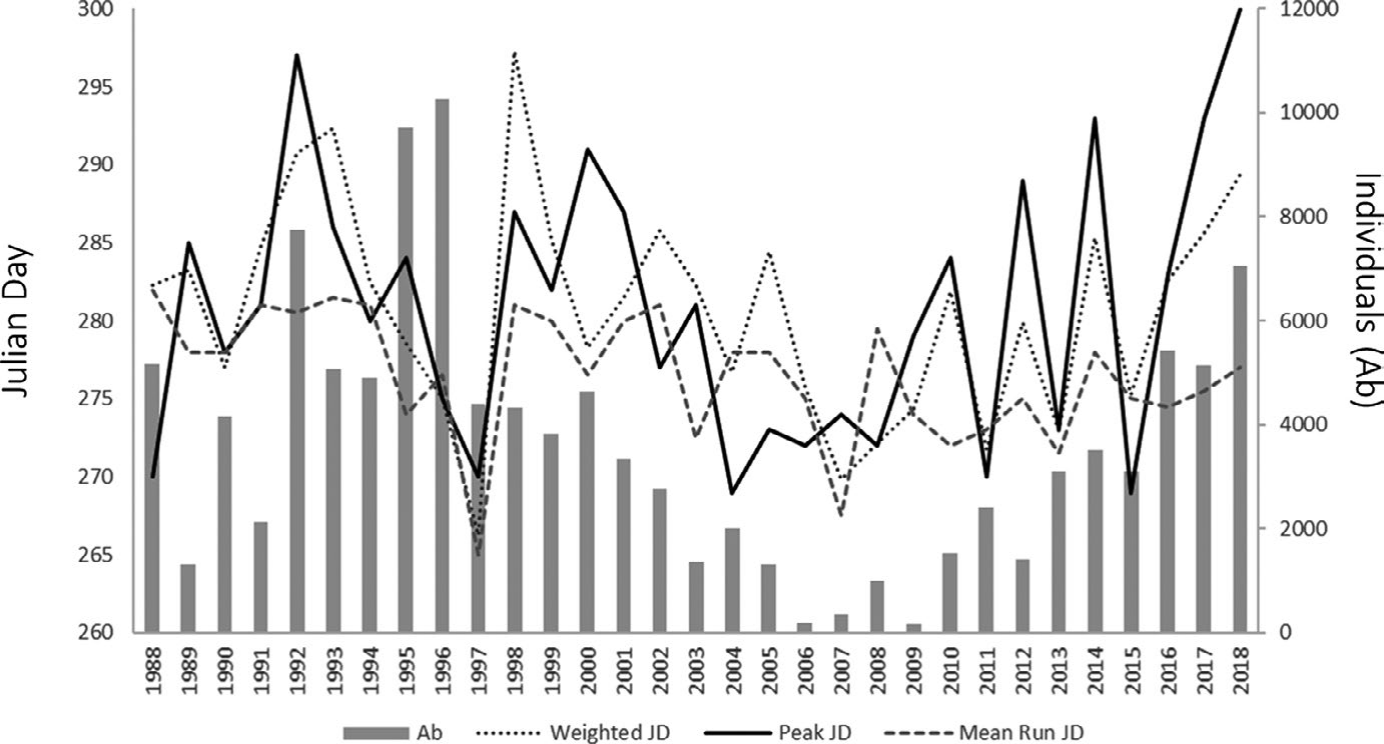
Hydrological and abundance trends. Weighted average Julian day of Chinook salmon counts by year at the Cowichan River fence operated by Fisheries and Oceans Canada, the Julian day where the peak migration occurs, and the mean run Julian day considering the beginning and end of the run independent of the number of counts (2000–2018). Abundance (Ab) and spawning migration time data were provided by the DFO South Coast stock assessment section (K. Pellet, unpublished data). Hydrological data (Station 08HA002) were from the Water Survey of Canada (2019)

Mean Chinook salmon run timing by Julian day, independent of the run size, has not changed significantly from the historical averages (Figure 3). However, peak spawning is happening later in the year with a marked shift in the mid 2000s. The latest historical peak on record occurred in 2018 (Julian day 300–October 26). Although the times series exhibit a trend for the peak spawning to occur later, historical values are seen as late as October 23 in 1992 and October 17 in 2000. The years 2014 to 2019 had September record low discharge levels after 2000 with a marked downward trend measured at the upper station (WSC 08HA002). The average river discharge for that month from 2014 to 2018 was 6.23 m^3^/s, lower than the 10.02 m^3^/s historical average post weir installation. Some of the lowest discharge values in the past 20 years occurred in 2014 and 2016 (4.73 and 4.62 m^3^/s, respectively).

## 4 DISCUSSION

### 4.1 Analytical considerations in hybrid identification

We have documented the repeated occurrence of natural hybridization between Chinook and Coho salmon. The frequency of hybrid occurrence varied depending on context (0.57% independently of their origin and 4.92% assuming origin in the Cowichan River). Given the opportunistic nature of this study, these values can only give us a general idea of hybrid presence, because the surveys in which hybrids they were encountered were not originally designed to target hybridization rates, lacking control variables and in‐river sampling. Despite this caveat, the hybridization observed may be an underestimate, as some hybrid fish in the surveys may have been identified as pure species Coho salmon, which were not subject to GSI.

Although the hybridization observed in this study is most likely natural, it was important to note that neither all Cowichan Chinook salmon broodstock were genotyped nor all the hatchery fish were marked or coded wire tagged (in recent years an average of 90%); therefore, human error is not entirely out of the question. However, given the reoccurrence of hybridization in multiple years, the number of parents involved, and that no hybrid or Coho salmon were genetically identified in the screened of hatchery broodstock, the observed hybridization remains very likely a nonhuman‐mediated event.

Although we intended to investigate if hybrids confirmed by molecular techniques could be identified by consistent meristic traits for rapid identification, due to the small sample size and the release of animals, our findings remain preliminary. Most hybrids were identified by genetic means among nonlethally sampled presumed Chinook salmon. Some hybrids could be identified visually by a general appearance intermediate between the parental species or by a disordered arrangement of scales on the caudal peduncle and associated lateral line deflection or disappearance (Figure 2). The pyloric caeca count for the single dissected hybrid (Fish 62, Table 5) was intermediate to the ranges reported for Coho and Chinook salmon by Clemens and Wilby (1961) and consistent with a report of intermediate counts in three natural Chinook–Coho salmon hybrids from a Lake Ontario tributary (Johnson & Ringler, 1981). A similar intermediate pyloric caeca count occurred in hybrids between lake trout and brook trout (Scott, 1967). Morphological and meristic characteristics of salmonid hybrids may be intermediate to, equal to, or exceed parental values and do not always provide reliable hybrid identification (Scribner et al., 2001).

### 4.2 Mechanism of hybridization

We suggest that the observed hybridization has been facilitated by prolonged low water levels in the Cowichan River, which limit habitat and promote later peak spawning of Chinook salmon, allowing the two species to cospawn.

Summer water levels in the Cowichan River watershed have declined significantly from the 1960s to the present as a result of increasing air temperatures, climatic shifts, and human pressures (Cowichan Valley Regional District, 2017; Spittlehouse, 2017). The number of days with low flows (below the 25th percentile) has increased nearly twofold from 1965 to 2015 (Pike et al., 2017). These low‐flow events are extending into September and October, during the historical peak Chinook salmon migration (Damborg et al., 2015).

The main stem of the Cowichan River is heavily utilized by spawning Coho salmon in addition to Chinook salmon, which are also occasionally observed in tributaries to the lake (K. Pellett, unpublished data). The upstream Chinook salmon migration peaks at the counting fence from late September to mid‐October, while spawning occurs from late October to early November. In contrast, Cowichan River Coho salmon spawn primarily from November through January (K. Pellett, unpublished data).

The peak spawning of Chinook salmon migration has shifted from late September in the mid‐2000s to late October in 2018. The brood year 2014 was the source of the majority of hybrids observed in the study and had the second lowest discharge value in the past 20 years. The change in peak spawning timing was correlated with a hydrological regime shift in the mid‐2000s, which likely involves a combination of climatic changes and transition to a warmer phase in the Pacific decadal oscillation and El Niño–Southern Oscillation (Newman et al., 2016). Other salmonid species have shown altered migration timing in response to climate change via water temperature and habitat availability (Crozier et al., 2011; Kovach et al., 2015). Surface water diversions for commercial and residential use have increased substantially over the last 65 years, with a threefold increase in the number of surface water licenses and a sixfold increase in groundwater wells (Cowichan Valley Regional District, 2017).

It is likely that habitat modifications have also played a role in reducing spawning grounds. Pike et al. (2017) provide an historical review of the Cowichan watershed alteration, which began with logging and the use of explosives to remove natural barriers in the early 20th century. Only five of the original 130 rapids remain on the main stem. Other habitat modifications included intensive logging practices, which reduced the capacity of watershed to balance hydrological processes, and the installation of a weir at the outlet of Cowichan Lake in 1956 for water storage purposes. There is limited hydrological monitoring in smaller streams, but anecdotal evidence supports the possibility that a lack of water availability during spawning reduces habitat causing the two species to overlap more than they did historically. Climate change that alters the habitat available for reproduction may sponsor hybridization between species that previously shared watersheds with little interaction (Garcia de Leaniz & Verspoor, 1989; Muhlfeld et al., 2009; Young et al., 2016).

Mitochondrial DNA indicated that the majority of hybrids were the result of female Coho salmon spawning with Chinook salmon males (all but two individuals from a reciprocal cross). This finding suggested two possible scenarios: accidental fertilization in crowded spawning grounds or heterospecific choice of mate when conspecifics are not available (e.g., differential abundance). If the available spawning habitat is very limited when the species overlap, it is possible that the hybrids are primarily the result of fortuitous fertilization from a “milt cloud” formed by an abundance of jack Chinook salmon attempting sneak fertilizations with Coho salmon females. However, differential abundance is a more plausible explanation.

Chinook salmon abundance in the Cowichan River drainage has increased in recent years. The abundance continues to recover from a record low of 540 adult natural spawners in 2009; 13,975 Chinook salmon were present in 2018. Cowichan River Chinook salmon are supplemented by hatchery production, but the level of hatchery production and proportion of hatchery fish in the watershed has declined over the past decade. The opposite is true for Coho salmon. Limited assessment work indicates that 20,000 individuals or less may now return to a system that once supported 10 times more (K. Pellet, unpublished data). Although Coho salmon could be relatively more abundant than Chinook salmon throughout the watershed, its abundance may be lower in spawning grounds traditionally shared by both species, such as the river section adjacent to the lake outlet.

A reproductive “bottleneck” may be experienced by Coho salmon females as they attempt reproduction when no conspecific males are available. This phenomenon is characteristic of the depensation or positive Allee effect and commonly observed in animal populations when their numbers dwindle (Gascoigne & Lipcius, 2004; Stephens et al., 1999). McKenzie et al. (2021) documented that females of both species preferentially mated with conspecific males, but would mate with a heterospecific male in the absence of a conspecific choice. Their study also found that the observed differences between fertilization rates of Chinook and Coho salmon eggs by males of either species were not significant, concluding there was little postzygotic reduction in embryonic viability. This finding suggests that the mechanism of hybridization is an incomplete reproductive barrier between these sister species.

### 4.3 Ecological concerns of hybrid occurrence

We report the existence of eight Chinook–Coho hybrid adults as well as the first F2+ juveniles encountered in the natural environment. In the case of the F2+ juveniles, the number of loci used in this study provides only a probabilistic estimation of their generation. One of these fish had a genotype compatible with being the offspring of an F1 by F1 mating. These findings, if substantiated in further analysis, raise the possibility of ongoing introgression within the Cowichan watershed. Recurrent episodes of interspecific hybridization may pose significant risks to the populations of both species, though Coho salmon may be at greater risk given the directional hybridization observed. If hybridization extends beyond the first generation, further potential impacts may include an expected loss of fitness in higher order hybrids due to the breakdown of species‐specific coadapted gene complexes; hybrids that may no longer possess a complete haploid genome of either species (Muhlfeld et al., 2009).

Hybridization that leads to introgression is not uncommon in salmonids (Allendorf et al., 2001; Scribner et al., 2001) and may have a variety of outcomes ranging from self‐limiting low levels of hybridization with restricted geographical penetration as has been documented for the genus *Salvelinus* (Gruzdeva et al., 2018; Hagen & Taylor, 2001) to the loss of both species in a “hybrid swarm” (Forbes & Allendorf, 1991; Young et al., 2016). While hybridization can be a relevant source of new variability that could lead to improved fitness or the development of new taxa (Abbott et al., 2013; Verspoor & Hammart, 1991), it can also be responsible for extirpation and extinction (Allendorf et al., 2001; Rhymer & Simberloff, 1996).

Given the potential negative impacts of hybridization, identification of hybrid fish extends beyond scientific curiosity. Hybrid occurrence can be used as a monitoring tool of ecosystem changes and determining its origin with certainty warrants the value of a large‐scale genetic monitoring program. Such program can help estimate hybrid frequency and assess the conditions under which they appear. The multiple and continuous years of hybridization observed in this study, along with the findings by McKenzie et al. (2021), suggest the frequency of hybridization between the two species may be more common than previously presumed. Examining why the hybridization between the two salmonid species is not observed more frequently in natural environments could guide subsequent studies.

## 5 CONCLUSION

Although presumed infertile and extremely rare, 63 Chinook–Coho salmon hybrids were encountered in a variety of surveys conducted over a 4‐year period in the vicinity of the Cowichan River. Among these hybrids, we report the existence of eight adults, as well as the first F2+ hybrid juveniles encountered in the natural environment. Although a targeted research program will be required to identify both the causative factors and true prevalence of hybrids among Chinook and Coho salmon, we provide evidence that implicates freshwater habitat modification resulting from climatic and human pressures in the increased cospawning of the two species. This overlap in spawning may have both spatial and temporal components arising from reduced habitat due to extended summer low‐flow periods. In addition, differential abundance between recovered levels of Chinook salmon and Coho salmon at historical lows may be leading to density‐dependent hybridization. The likelihood of ongoing warming temperatures and drier summers in watersheds of southern BC combined with increasing human pressures on watersheds indicate that interspecific salmonid hybridization and introgression may be observed more frequently in the future, which warrants the value of hybrid identification as an ecosystem monitoring tool within a large‐scale survey program.

## CONFLICT OF INTEREST

The authors declare no conflict of interest.

## AUTHOR CONTRIBUTION

**H. Andres Araujo:** Conceptualization (lead); Formal analysis (equal); Investigation (lead); Methodology (equal); Supervision (lead); Writing‐original draft (lead). **William D. P. Duguid:** Investigation (lead); Methodology (equal); Validation (equal); Visualization (equal); Writing‐original draft (equal). **Ruth E Withler:** Formal analysis (equal); Investigation (equal); Methodology (equal); Validation (equal); Writing‐original draft (equal). **Angela D. Schulze:** Data curation (equal); Formal analysis (equal); Investigation (equal); Methodology (equal). **Janine Supernault:** Formal analysis (equal); Methodology (equal). **Jessica L. McKenzie:** Investigation (equal); Validation (equal). **Kevin Pellett:** Data curation (equal); Investigation (supporting). **Terry D. Beacham:** Investigation (equal); Resources (equal); Writing‐review & editing (supporting). **Kim Jonsen:** Investigation (equal); Validation (equal); Visualization (equal). **Anna Gummer:** Data curation (supporting); Methodology (supporting); Writing‐review & editing (supporting).

## Supporting information

Appendix S1

## Data Availability

Supplementary data are available in this document as an Appendix S1 and through DRYAD.
