# Supplementary material for: Chinook and Coho salmon hybrids linked to habitat and climatic changes on Vancouver Island, British Columbia
Source: Ecol Evol. 2021 Nov 11;11(23):16874–89. doi: 10.1002/ece3.8322 (PMC8668772; doi:10.1002/ece3.8322)
Supplement: Supplementary file 1 — Appendix S1 [file ECE3-11-16874-s001.docx]

**Appendix**

Table 1.S. Mitochondrial DNA Assay (simplified version). For the full assay please see Dryad, link provided in the Data Accessibility Statement.

| **Paper Fish #** | **Paper Survey name** | **MtDNA** | **NGS-16S** | **NGS-COISal** |
| --- | --- | --- | --- | --- |
| **1** | DFO Juvenile Trawl | Coho | Coho | Coho |
| **2** | DFO Juvenile Trawl | Coho | Coho | Coho |
| **3** | Puget Sound Juveniles | no result | reads too low | no result |
| **4** | Area 121 Recreational | Coho | #N/A | #N/A |
| **5** | DFO Juvenile Seine | Coho | Coho | Coho |
| **6** | DFO Juvenile Seine | Coho | Coho | Coho |
| **7** | DFO Juvenile Seine | Coho | Coho | Coho |
| **8** | DFO Juvenile Seine | Coho | Coho | Coho |
| **9** | DFO Juvenile Seine | Coho | Coho | Coho |
| **10** | DFO Juvenile Seine | Coho | Coho | Coho |
| **11** | DFO Juvenile Seine | Coho | Coho | Coho |
| **12** | Juvenile Marine Tagging | Coho | Coho | Coho |
| **13** | Juvenile Marine Tagging | Coho | Coho | Coho |
| **14** | Juvenile Marine Tagging | Coho | Coho | Coho |
| **15** | Juvenile Marine Tagging | Coho | Coho | Coho |
| **16** | Juvenile Marine Tagging | Coho | Coho | Coho |
| **17** | Juvenile Marine Tagging | Coho | Coho | Coho |
| **18** | Juvenile Marine Tagging | Coho | Coho | Coho |
| **19** | Juvenile Marine Tagging | Coho | Coho | Coho |
| **20** | Juvenile Marine Tagging | Coho | Coho | Coho |
| **21** | Juvenile Marine Tagging | Coho | Coho | Coho |
| **22** | Juvenile Marine Tagging | no result | reads too low | reads too low |
| **23** | Juvenile Marine Tagging | no result | reads too low | reads too low |
| **24** | Juvenile Marine Tagging | no result | reads too low | reads too low |
| **25** | Juvenile Marine Tagging | no result | reads too low | reads too low |
| **26** | Juvenile Marine Tagging | no result | reads too low | reads too low |
| **27** | Juvenile Marine Tagging | no result | reads too low | reads too low |
| **28** | Juvenile Marine Tagging | no result | reads too low | reads too low |
| **29** | Juvenile Marine Tagging | no result | reads too low | reads too low |
| **30** | Juvenile Marine Tagging | no result | reads too low | reads too low |
| **31** | Juvenile Marine Tagging | no result | reads too low | reads too low |
| **32** | Juvenile Marine Tagging | no result | reads too low | reads too low |
| **33** | Juvenile Marine Tagging | no result | reads too low | reads too low |
| **34** | Robertson R. | Chinook | Chinook | Chinook |
| **35** | Robertson R. | Chinook | Chinook | Chinook |
| **36** | Area 19/20 Recreational | ambiguous | Coho (53%)+Chinook (47%) | Coho (53%)+Chinook (47%) |
| **37** | DFO Juvenile Seine | Coho | Coho | Coho |
| **38** | DFO Juvenile Seine | Coho | Coho | Coho |
| **39** | Juvenile Marine Tagging | Coho | Coho | Coho |
| **40** | Juvenile Marine Tagging | Coho | Coho | Coho |
| **41** | Juvenile Marine Tagging | Coho | Coho | Coho |
| **42** | Juvenile Marine Tagging | Coho | Coho | Coho |
| **43** | Juvenile Marine Tagging | Coho-low detection | reads too low | Coho |
| **44** | Juvenile Marine Tagging | no result | reads too low | reads too low |
| **45** | Juvenile Marine Tagging | no result | reads too low | reads too low |
| **46** | Area 1 Recreational | Coho | Coho | Coho |
| **47** | Area 125 Recreational | Coho | Coho | Coho |
| **48** | Area 17 Recreational | Coho | Coho | Coho |
| **49** | Area 19/20 Recreational | Coho | Coho | Coho |
| **50** | Area 27 Recreational | Coho | Coho | Coho |
| **51** | DFO Juvenile Seine | #N/A | #N/A | #N/A |
| **52** | DFO Juvenile Seine | #N/A | #N/A | #N/A |
| **53** | Juvenile Marine Tagging | Coho | Coho | Coho |
| **54** | Juvenile Marine Tagging | Coho | Coho | Coho |
| **55** | Juvenile Marine Tagging | Coho | Coho | Coho |
| **56** | Juvenile Marine Tagging | Coho | Coho | Coho |
| **57** | Juvenile Marine Tagging | Coho | Coho | Coho |
| **58** | Juvenile Marine Tagging | Coho | Coho | Coho |
| **59** | Juvenile Marine Tagging | Coho | Coho | Coho |
| **60** | Juvenile Marine Tagging | Coho | Coho | Coho |
| **61** | Juvenile Marine Tagging | Coho | Coho | Coho |
| **62** | Juvenile Marine Tagging | Coho | Coho | Coho |
| **63** | Adult Marine Tagging | #N/A | Coho | Coho |

Table 2.S. SNP amplicons results. Ab=Absent; He=Heterozygous; NC=No Call

| **Fish #** | **SNP Amplicons** | | | | |
| --- | --- | --- | --- | --- | --- |
|  | **OkiOts113** | **OkiRad45** | **OkiRad51** | **OkiRad195** | **OkiRad198** |
| **1** | He | He | He | He | He |
| **2** | NC | NC | NC | NC | He |
| **3** | NC | NC | NC | NC | He |
| **4** | He | He | He | He | He |
| **5** | He | He | He | He | He |
| **6** | He | He | He | He | He |
| **7** | He | He | He | He | He |
| **8** | He | He | He | He | NC |
| **9** | He | He | He | He | He |
| **10** | He | He | He | He | He |
| **11** | He | He | He | He | He |
| **12** | He | He | He | He | He |
| **13** | He | He | He | He | He |
| **14** | He | He | He | He | He |
| **15** | He | He | He | He | NC |
| **16** | He | He | He | He | He |
| **17** | He | He | He | He | He |
| **18** | He | He | He | He | He |
| **19** | He | He | He | He | He |
| **20** | He | He | He | He | He |
| **21** | He | He | He | He | He |
| **22** | He | He | He | He | He |
| **23** | He | He | He | He | He |
| **24** | He | He | He | He | He |
| **25** | NC | He | He | He | He |
| **26** | NC | NC | NC | NC | NC |
| **27** | He | NC | NC | He | He |
| **28** | He | NC | NC | NC | He |
| **29** | He | NC | NC | NC | He |
| **30** | He | He | He | He | He |
| **31** | He | He | He | He | He |
| **32** | NC | NC | NC | NC | He |
| **33** | He | He | He | He | NC |
| **34** | NC | NC | NC | Ab | Ab |
| **35** | NC | NC | NC | Ab | Ab |
| **36** | He | He | He | He | NC |
| **37** | He | He | He | He | He |
| **38** | He | He | He | He | He |
| **39** | NC | NC | NC | NC | NC |
| **40** | He | He | He | He | He |
| **41** | He | He | He | He | He |
| **42** | NC | NC | NC | NC | NC |
| **43** | He | NC | NC | He | He |
| **44** | NC | NC | NC | NC | He |
| **45** | NC | NC | NC | NC | NC |
| **46** | He | He | He | He | NC |
| **47** | He | He | He | He | He |
| **48** | He | He | He | He | He |
| **49** | He | He | He | He | He |
| **50** | He | He | He | He | He |
| **51** | NC | NC | NC | NC | Ab |
| **52** | NC | NC | NC | NC | NC |
| **53** | He | He | He | He | He |
| **54** | He | He | He | He | NC |
| **55** | He | He | He | He | He |
| **56** | He | He | He | He | He |
| **57** | He | He | He | He | He |
| **58** | He | He | He | He | He |
| **59** | He | He | He | He | NC |
| **60** | He | He | He | He | He |
| **61** | He | He | He | He | He |
| **62** | NC | NC | NC | NC | He |
| **63** | He | He | He | He | He |
